# Supplementary figures and images for: The HDAC9-associated risk locus promotes coronary artery disease by governing TWIST1
Source: PLoS Genet. 2022 Jun 17;18(6):e1010261. doi: 10.1371/journal.pgen.1010261 (PMC9246173; doi:10.1371/journal.pgen.1010261)

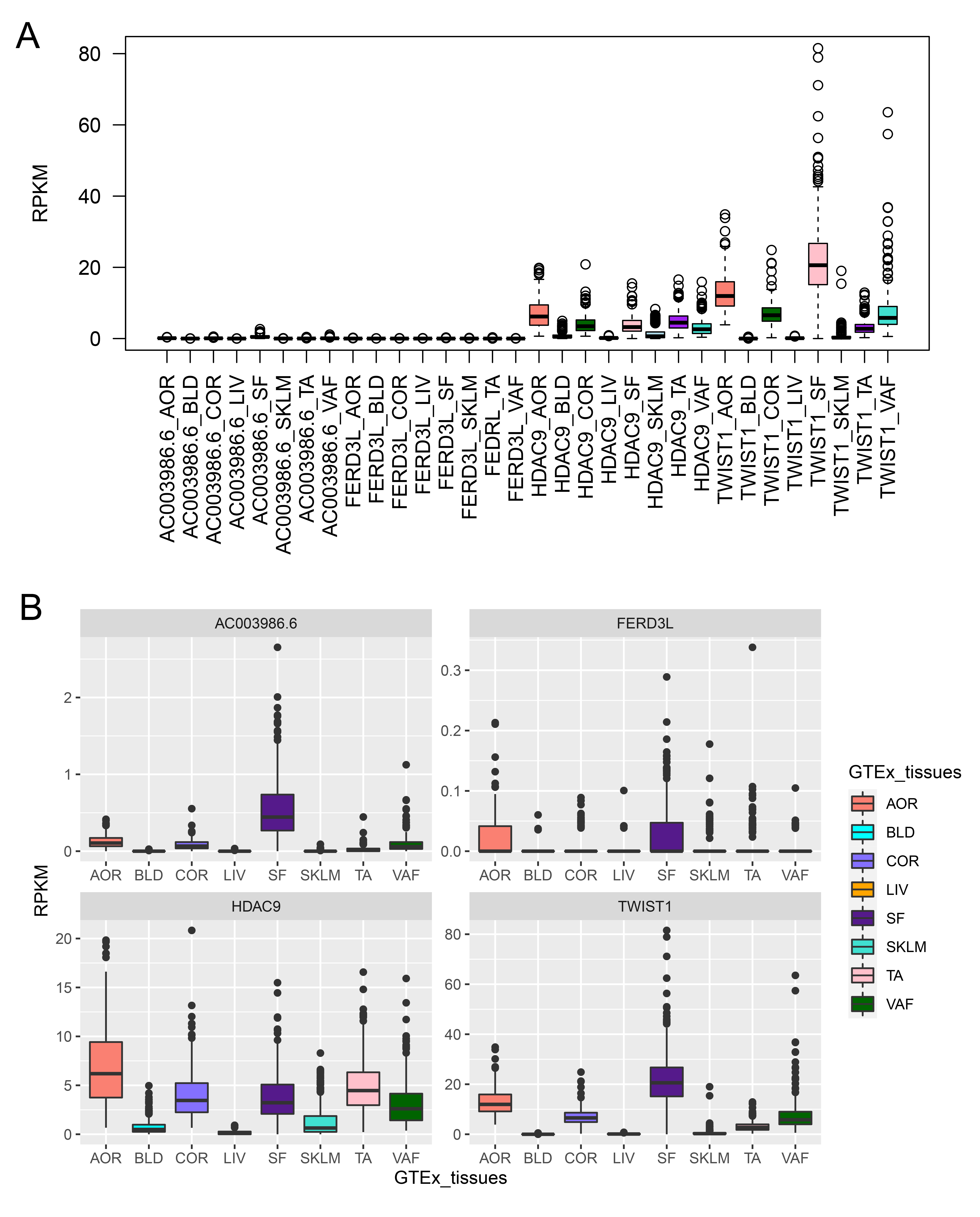

Supplement: S1 Fig — (A) Gene expression levels of HDAC9, TWIST1, FERD3L and AC003986.6 in GTEx–generally showing higher expression levels of HDAC9 and TWIST1 across various tissue subtypes. (B) Same as A but at different scales for each gene. RPKM, Reads Per Kilobase of transcript per Million mapped reads. (TIF) [file pgen.1010261.s001.tif]

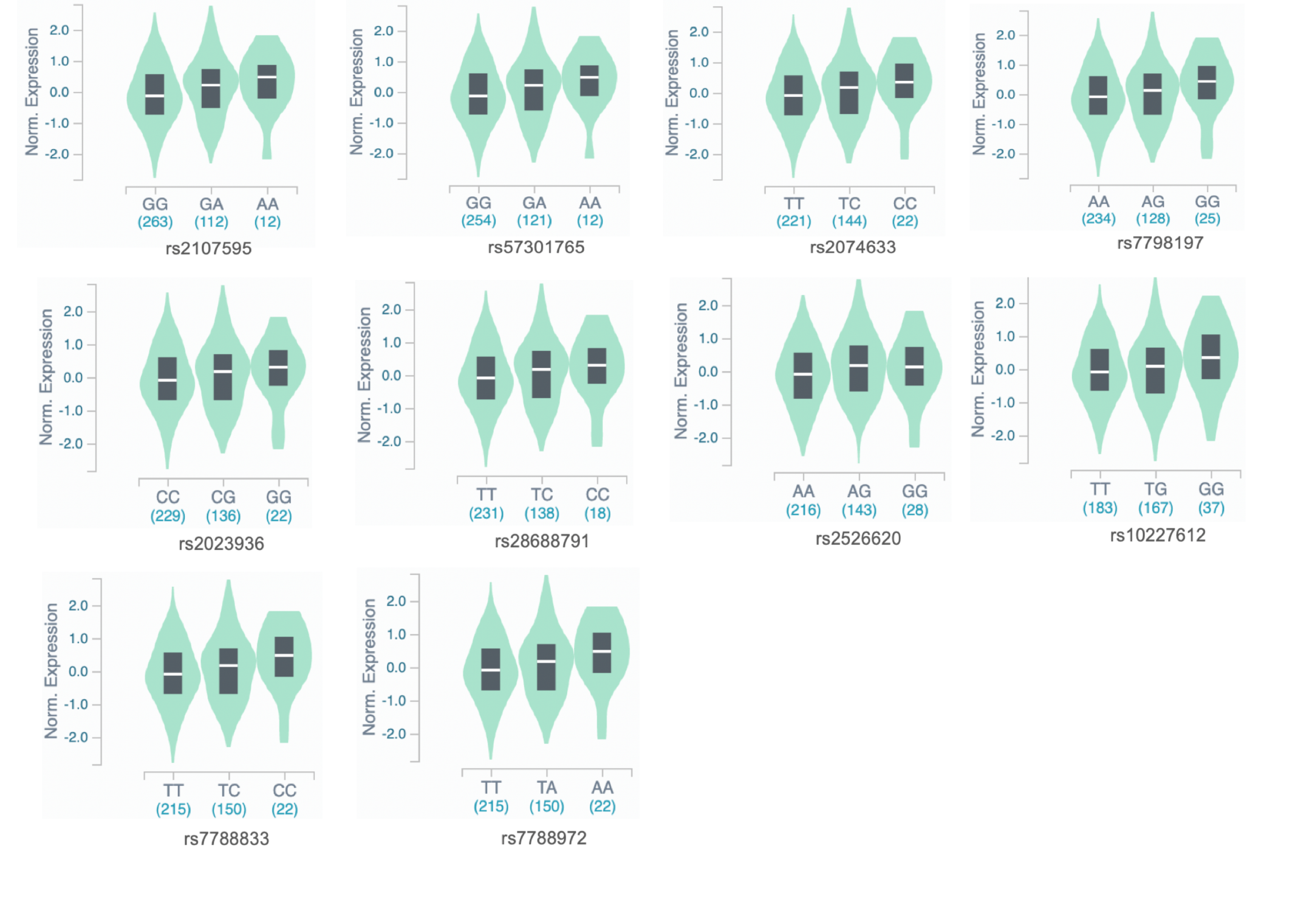

Supplement: S2 Fig — eQTL violin plots are shown for tissue expression of TWIST1 in AOR in GTEx corresponding to SNPs in the HDAC9-associated CAD risk locus. The CAD risk allele is presented on the right in all panels, indicating that the CAD risk allele is consistently associated with higher expression levels of TWIST1 in AOR. There are no FDR or adjusted P value considerations made in GTEx (this is not possible through the GTEx website). This Figure corresponds to S2 Table. (TIF) [file pgen.1010261.s002.tif]

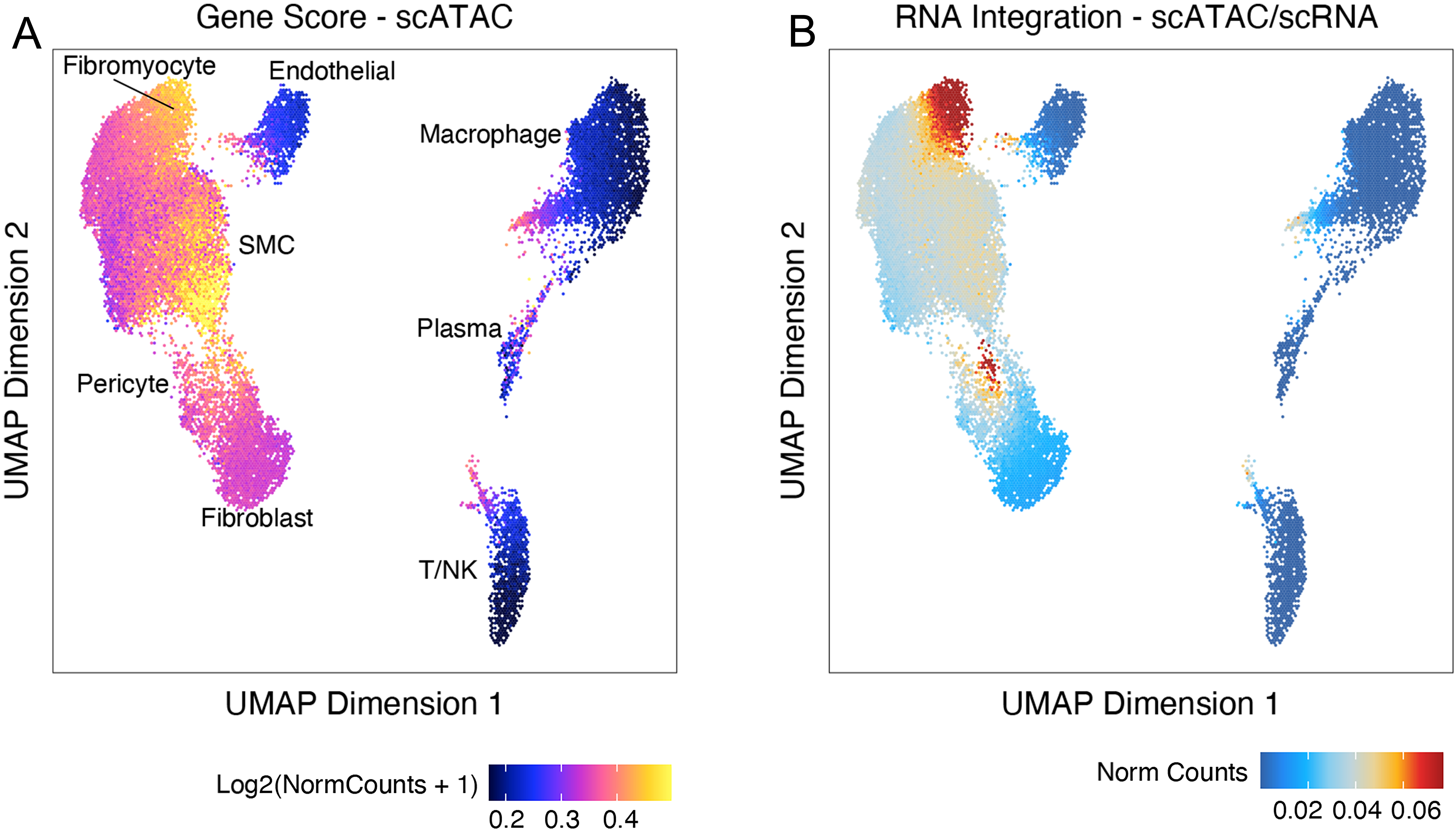

Supplement: S3 Fig — (A) UMAP plot showing the greater chromatin accessibility in smooth muscle cells (SMC) and fibroblasts relative to other cell types for TWIST1. (B) UMAP feature plot from integrated scATAC/scRNA-seq data in coronary artery, which more clearly shows TWIST1 expression in modulated SMCs annotated as fibromyocytes. Labelling of annotated clusters is based on top defining markers from Turner et al. [45]. (TIF) [file pgen.1010261.s003.tif]

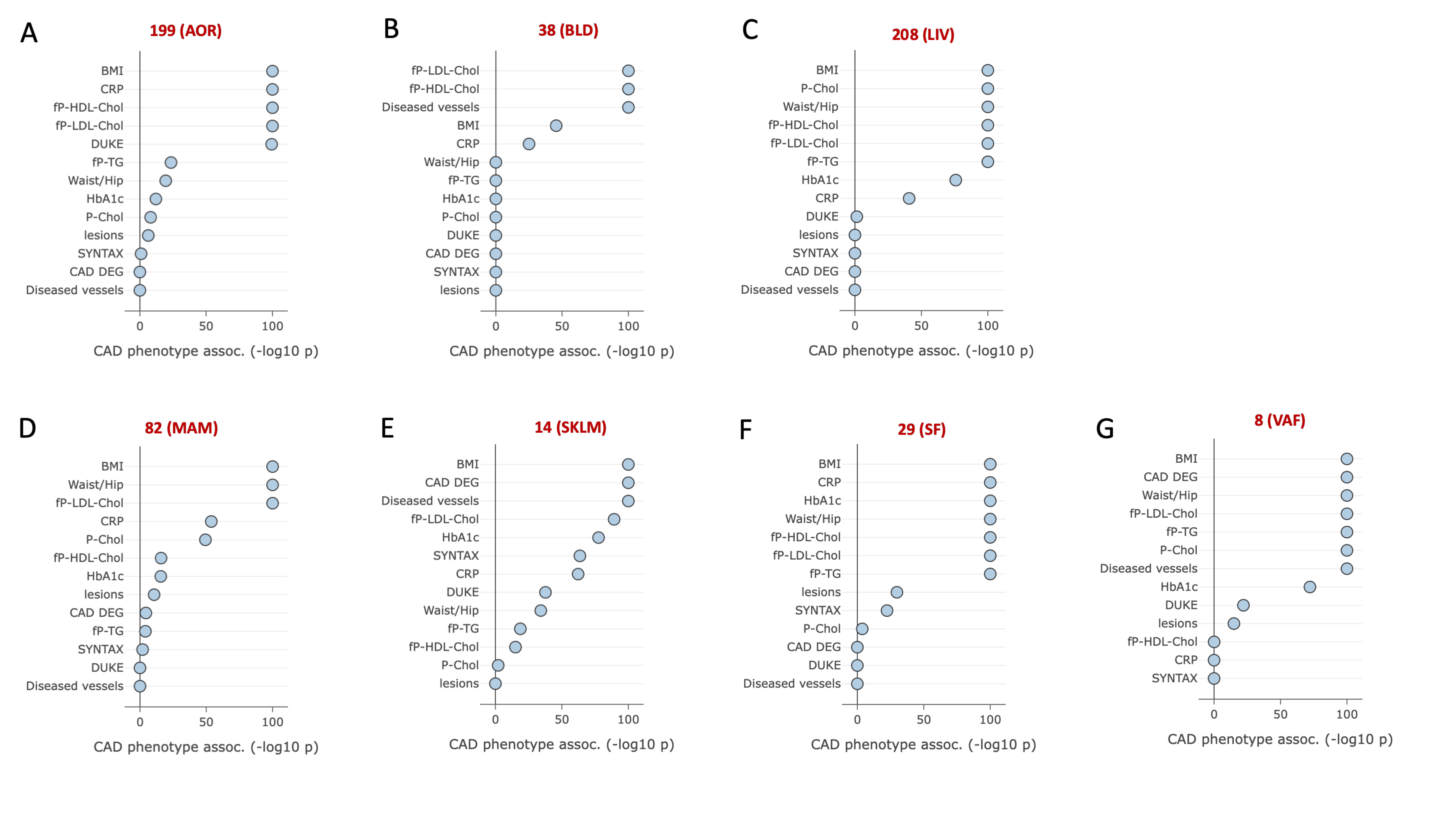

Supplement: S4 Fig — Shown here are the 7 GRNs that involve HDAC9 (GRNs 8, 14, 29, 38, 82, 199, 208) and their associations with clinical traits in the STARNET study. The tissue in which HDAC9 is a member of each of these GRNs is shown in brackets (e.g. 199 (AOR), signifies that HDAC9 expressed in the Aorta participates in GRN 199). With the exception of GRN 208, each of these was associated with the severity of CAD as assessed by number of coronary lesions (“Lesions”), number of diseased vessels (“Diseased vessels”), SYNTAX score (“SYNTAX”) or Duke CAD index (“DUKE”). These data are also available from the dedicated STARNET website described in Koplev et al (starnet.mssm.edu) [10]. (TIF) [file pgen.1010261.s004.tif]
