# Supplementary material for: The HDAC9-associated risk locus promotes coronary artery disease by governing TWIST1
Source: PLoS Genet. 2022 Jun 17;18(6):e1010261. doi: 10.1371/journal.pgen.1010261 (PMC9246173; doi:10.1371/journal.pgen.1010261)
Supplement: Attachment — Submitted filename: Response to reviewers.docx [file pgen.1010261.s008.docx]

**Response to Reviewers**

**Reviewer #1**

**Reviewer #1:** In this manuscript, Ma and colleagues present their analysis of the causal variation and gene responsible for the CAD association at the 7p21.1 locus. This is an important question, as this locus is highly associated with multiple vascular diseases, and there is controversy regarding the causal gene at this locus, with some studies pointing to HDAC9 and others to TWIST1. The authors embark on a comprehensive suite of analyses to identify the most likely causal SNP and the causal gene, which they conclude to be the lead SNP rs2107595 and TWIST1, respectively. The analyses of the locus architecture, and the eQTL-based analyses of gene causality are solid. In particular, the human single cell ATAC data contributes new, complementary evidence that strengthens the linkage between rs2107595 and TWIST1. The study also fails to replicate the previously reported eQTL for HDAC9 in inflammatory cells, which is potentially noteworthy. However, other analyses (eQTL, Mendelian Randomization) have been previously done for this locus, also with using STARNET data, and have reached similar conclusions. There are also significant issues with the TWIST1 correlation and GRN analyses that limit their interpretability. However, this study does provide important incremental evidence to support the assertion that TWIST1 is the causal gene at this important locus.
**Response:** We thank Reviewer #1 for their positive comments regarding our manuscript. We note certain criticisms raised in the paragraph above are reiterated and expanded upon in the specific comments below. We therefore address these concerns below.

MAJOR COMMENTS:

**Reviewer #1:** A very similar mendelian randomization analysis has previously been performed for TWIST1 and CAD, with a significant MR association between TWIST1 and CAD using the same resource (STARNET mammary artery/internal thoracic artery, [https://doi.org/10.1016/j.ajhg.2017.04.016](https://protect-au.mimecast.com/s/5SH3Cp81PvhED1LS2NYhi?domain=doi.org), supplemental table 3), rendering the current eQTL-based analyses largely redundant/confirmatory.

**Response:** Thank you for this comment. The analysis and result referred to by Reviewer #1 in Hauberg (2017) are very well known to us, because several of our co-authors were co-authors on that manuscript. We can summarize the Hauberg et al paper by stating that it was a very large-scale study which integrated associations of common genetic variants in 57 GWASs with 24 studies of expression quantitative trait loci (eQTLs) from a broad range of tissues by using a Mendelian randomization (MR) approach. We note that the specific result referred to by Reviewer #1 does not actually appear in the relevant main manuscript, but as a single line in Supplementary Table 3 in that paper. To be precise, it appears as line 1682 in Supplementary Table 3 (that has a total of 9657 lines).

The Hauberg et al MR analysis was totally unfocussed and studied 57 GWASs with 24 studies of eQTLs from a broad range of tissues and 30-40 different diseases and phenotypes. In contrast, our study is sharply focused on the HDAC9-associated risk locus, with a single endpoint (CAD) and 2-samples MR only. As such, Hauberg et al (2017) were not able to test for the causal effect of rs2107595 in all STARNET tissues, because of the need to account for genome-wide significant thresholds in the eQTL datasets which were necessary for their analysis. In contrast, our focused analysis for the current manuscript was able to undertake systematic testing of rs2107595 in all available STARNET tissues at more relaxed cis-eQTL significance cutoffs due to the lack of multiple comparison testing. As another key point of difference, in the current study we used updated datasets – that included both updated and larger GWAS datasets and also updated STARNET datasets. Specifically, Hauberg et al did not include macrophage and foam cell data from STARNET.

As yet another key point of difference, Hauberg et al found that TWIST1 was causal for CAD in the Internal Mammary Artery (referred to in Hauberg et al as Internal Thoracic Artery (ITA)). This is a different finding to the current submission, where we found TWIST1 was causal for CAD in Aorta. While both are arterial tissues, we consider that all of the above reasons have allowed this more updated and refined result to now emerge in the current study. The fact that we have found TWIST1 to be causal for CAD in Aorta is a more logical result, because while the Aorta is strongly predisposed to developing atherosclerosis, the Internal Mammary Artery is relatively protected from developing this disease.

Finally, the current submission applies a series of distinct but inter-related informatic tools to study the HDAC9-associated risk locus, with MR being only one of many analyses conducted. For example our submission also includes studies of linkage disequilibrium relationships; HDAC9 levels, structure and function; scATAC-seq; and gene regulatory networks. This comprehensive approach is important and necessary to explore the full complexity of this risk locus – which was obviously not done at all in Hauberg et al.

While these points clearly highlight the very major differences and lack of redundancy between the studies, we acknowledge that it was an omission not to have mentioned and cited the Hauberg et al study. Accordingly, the following text now appears in the introduction:

*“In addition, both a large-scale mendelian randomization analysis and an integrative genomics analysis suggested that TWIST1 is potentially the causal CAD gene related to this locus, and that this causal effect of TWIST1 is operative in the arterial wall.”*

**Reviewer #1:** Regarding the correlation of TWIST1 expression levels to other genes in the artery wall, the finding that 16,472 genes were ‘significantly’ associated with TWIST1 expression despite Bonferroni correction is difficult to fathom, given there are only ~20,000-25,000 genes in the human genome. In addition, there is a strong chance that many of these associations are simply driven by cell type - i.e. if TWIST1 is expressed highly in a cell type within the bulk RNAseq data, all other genes in that cell type will be highly correlated only by virtue of their elevated expression in that cell type, not because they are in a pathway with TWIST1.

**Response:** We thank Reviewer #1 for this insightful comment. We do not disagree with this comment and after consideration we felt the most appropriate step was to remove these data from the manuscript.

**Reviewer #1:** Fig. 7G - the GO processes presented here are extremely generic; it is difficult to ascribe any specific role to TWIST1 using these GO terms. This may be related to including too many ‘significant’ gene associations as per the above comment.

**Response:** As per the comment above, we elected to remove Figure 7 from the manuscript entirely.

**Reviewer #1:** The authors infer that the presence of HDAC9 in 7 GRNs ‘confirms’ that HDAC9 is likely involved in the pathogenesis of CAD - to support this statement, we would need to know whether the GRNs in which HDAC9 resides are also associated with CAD phenotypes, similar to the TWIST1-containing GRNs.

**Response:** Thank you for this suggestion. We agree it is important to provide these data to substantiate this claim. We have added a new Figure (Supplementary Figure 4) that shows the phenotypic associations for all 7 of the GRNs that include HDAC9. Importantly, and supporting our comments that HDAC9 is involved in the pathogenesis of CAD, 6 out of the 7 GRNs that include HDAC9 are associated with the severity of CAD.

**Reviewer #1:** Fig. 8 - the use of multiple different tissues to form GRNs is problematic in my mind, for a similar reason that I object to correlation of TWIST1 with other transcripts from bulk tissue samples containing multiple cell types. For example, gene expression will vary dramatically between tissues, causing strong correlations in the GRNs, but most often these genes do not actually have a real regulatory relationship and instead are merely correlated across tissues.

**Response:** Thank you for this comment, although, we most respectfully disagree. The inferring of GRNs from STARNET was a very major undertaking that spanned several years. At the time of first submission of this manuscript regarding TWIST1/HDAC9 to PLOS genetics, the full STARNET GRN results had not been published. However, we are pleased to inform Reviewer #1 that the full methods and results of the GRN analyses were published in January 2022 in the inaugural edition of Nature Cardiovascular Research (Koplev et al. A mechanistic framework for cardiometabolic and coronary artery diseases. Nature Cardiovascular Research. 2022;1:85-100). Accordingly, we now cite this manuscript in the current submission. As to be expected, this study at Nature Cardiovascular Research where we inferred gene networks across tissues underwent very rigorous peer review and vetting of the methodologies. Therefore, we most respectfully suggest that this comment relating to our published and accepted GRN methodologies lies beyond the scope of what we can reasonably address with respect to the current submission to PLOS genetics.

MINOR COMMENTS:

**Reviewer #1:** I am not sure that a ‘pipeline’ is the best way to describe the set of analyses in this manuscript. A pipeline often refers to processing of data in an end-to-end fashion with minimal user supervision outside of the adjustment of parameters. This manuscript describes a collection of discrete analyses, each requiring intensive human interpretation.

**Response:** Thank you for this comment. We gave significant consideration as to how best to respond. We appreciate that the term ‘pipeline’ has certain implications as Reviewer #1 has indicated. However, in considering how best to respond we felt somewhat constrained by the fact that in our original description of this informatics approach (Ma et al. Atherosclerosis. 2020; 311:20-29), it was already labelled a pipeline. Below is the Graphical Abstract from that publication – included here to highlight that this has already been published as a “pipeline”, and also to demonstrate that despite the fact that human interpretation was required, the current manuscript on TWIST1/HDAC9 has remained absolutely faithful to end-to-end data processing steps in the ‘pipeline’ as originally described. Therefore, while we agree that significant human interpretation was required, we would also respectfully claim that we did indeed process the data in an end-to-end fashion and that outside of adjustment of parameters related to this locus there were minimal if any user adjustments made to the ‘pipeline’.

Given our dilemma that this has already been publicly published and described as a ‘pipeline’, and taking the above considerations into account, we felt the best approach was to acknowledge this in the limitations section. The initial part of the limitations section now reads as follows:

*“There are certain limitations of this study. First, since its description in 2020, we have continued to improve this pipeline and it is likely that future refinements will continue to increase its efficiency and accuracy. In addition, although we describe this approach as a ‘pipeline’ (which typically implies seamless end-to-end data processing with minimal user supervision), significant human interpretation of the results is required. It is hoped that this ‘pipeline’ approach to understand key risk loci can become less reliant on human interpretation as it evolves.”*

Furthermore, we have simplified the title of the manuscript and changed it from “*An Efficient Informatics Pipeline to Understand Complex Gene Loci: The HDAC9-associated risk locus promotes coronary artery disease by governing TWIST1*” now to simply: “The HDAC9-associated risk locus promotes coronary artery disease by governing TWIST1”

**Reviewer #1:** LD scores of the GWAS SNPs were calculated using 1000 Genome data - in what ancestry group(s)?

**Response:** Thank you. We used all ancestry groups and have amended the relevant text in the methods section that now states:

*“Linkage disequilibrium (LD) scores of the GWAS SNPs were calculated using 1000 Genome data with all ancestry groups, including African American, Hispanic, European and Asian, by LDlinkR in R (4.0.2).”*

**Reviewer #1:** Regarding GTEx tissues - why not also use tibial artery? Expected to have many common features with AOR/COR and may increase power

**Response:** Thank you for this excellent suggestion. We reran all relevant GTEx analyses and included Tibial Artery (TA). These data are now presented in revised Supplementary Figure 1 and Supplementary Table 2. Exactly as both we and Reviewer #1 would have expected, the TA results were similar to the prior GTEx and STARNET results presented using arterial tissues in this manuscript. Specifically, as shown in revised Supplemental Figure 1, the expression levels of HDAC9 and TWIST1 in TA were very similar to those in AOR and COR. Interestingly, there were no eQTLs in the HDAC9-associated CAD risk locus in TA, therefore, while we reran the analysis including TA it did not result in any changes to Supplementary Table 2, but we now mention in the legend to this table that TA is included in this analysis.

**Reviewer #1:** Given similar TWIST1 and HDAC9 expression in arterial tissues, the authors may want to explicitly state that the lack of a significant HDAC9 eQTL in these tissues cannot be explained by low HDAC9 levels.

**Response:** Thank you for this helpful suggestion. The following text has been added in the second paragraph of the discussion:

*“The lack of any significant eQTLs for HDAC9 cannot be explained by low gene expression levels, as these were comparable between HDAC9 and TWIST1 in all tissues (Figure 4, Supplementary Figure 1).”*

**Reviewer #1:** Regarding the lack of a significant eQTL relationship for HDAC9 in macrophages and foam cells, how many samples for each are present in the STARNET data, and what were the levels of HDAC9 in these samples?

**Response:** We appreciate this comment and agree this is important information.

With respect to the levels of HDAC9 in macrophages (MP) and foam cells (FC), these data were already in the manuscript in Figure 4B. These data remain in this revised version.

With respect to the number of samples, we have now included the exact number of subjects in each STARNET eQTL analysis for the entire study and added the following new text to the methods section of the manuscript:

*After the above steps, the following numbers of subjects were available for the final eQTL analysis for each tissue: AOR 515, MAM 520, LIV 523, SKLM 514, BLOOD 471, SF 550, VAF 509, FC 81, MP 96.”*

While there were fewer samples in the MP and FC groups in STARNET, it is critical to note the following points about the MP and FC analyses:

- The MP and FC groups were still a well powered eQTL analysis. For example, in a prior publication using our samples to generate eQTL data on the gene PHACTR1 (Kiando et al. PLoS Genet. 2016 Oct 28;12(10):e1006367), as seen in Figure 2B of that paper, with 86 subjects we achieved a P value of 0.003 for that eQTL analysis.
- It is also important to note that both MP and FC require ex vivo culture under rigorously standardized protocols, whereas all other STARNET samples are collected and processed fresh. The standardized MP and FC culture techniques have the effect of removing much of the normal biologic variability involved with human tissue collection – because the variability in subject-to-subject environmental differences are not present under standardized culture techniques. This removes ‘background noise’ from these samples.
- MP and FC are pure cell populations. All other STARNET tissues are heterogeneous. For example the AORTA samples from STARNET include endothelial cells, smooth muscle cells, fibroblasts, immune cells etc. It has been our very strong experience that the purity of the MP and FC populations in STARNET, combined with the removal of ‘background noise’ via the ex vivo standardized culture, provides much greater ability to resolve eQTLs than the other tissues despite the smaller sample size.
- A cursory look at our most recent paper using STARNET MP and FC data, Hao et al Circ Genom Precis Med. 2022;15:e003365, will rapidly affirm the above comments as it is immediately obvious that MP and FC are evenly represented among the differing tissues in the results of that paper.

We have refrained from adding these lengthy and tangential discussions to the manuscript related to the samples size of MP and FC, but we trust that per Reviewer #1’s original comment, it can now be appreciated that the MP and FC findings with respect to HDAC9 and eQTLs are absolutely robust.

**Reviewer #1:** Fig.6 - please include a UMAP of cell clusters from the scATAC data or scATAC/scRNA combined data in this figure. Top defining markers of each cluster should also be given.

**Response:** Thank you for this helpful suggestion. We have now included a UMAP feature plot showing the ArchR based imputed gene score activity for TWIST1 derived from the coronary artery scATAC data (Supplementary Figure 3A). This reflects the greater chromatin accessibility in smooth muscle cells and fibroblasts relative to other cell types. We have also provided a UMAP feature plot from the integrated scATAC/scRNA data in coronary artery, which more clearly shows TWIST1 expression in modulated smooth muscle cells annotated as fibromyocytes (Supplementary Figure 3B). We have labelled the annotated clusters based on top defining markers which are already presented in the manuscript in press (Turner AT et al Nat Gen 2022) and in a bioRxiv preprint - https://doi.org/10.1101/2021.06.07.447388.

**Reviewer #1:** Fig. 6 - what is the ‘coronary’ track? Is this a composite of all cell types?

**Response:** The ‘coronary’ track represents a bulk ATAC-seq library from 50,000 nuclei (therefore consisting of several cell types) generated from human coronary artery. Our recent study (Turner AT et al – reference 45 in the main manuscript) demonstrates that aggregating accessibility profiles from individual nuclei to generate ‘pseudobulk’ profiles strongly corresponds with bulk ATAC-seq accessibility profiles. We have added the following sentence to the Figure 6 legend to clarify what the ‘coronary’ track represents, as follows:

“*The ‘Coronary’ track (uppermost track in A) represents a bulk ATAC-seq library from 50,000 nuclei (therefore consisting of several cell types) generated from human coronary artery.^45^*”

**Reviewer #1:** Fig. 6 - From this view, there does not appear to be a great deal of open chromatin at the putative enhancer sites harboring rs2107595 and its LD SNPs. A zoomed in view of this region would be helpful to the reader. Also, given rs2107595 is the putative causal SNP, it would be helpful to see this SNP annotated in a different color vs the other SNPs.

**Response:** Thank you for this very useful suggestion. We have added a new zoomed in view (Figure 6B) showing open chromatin peaks and peak2gene links overlapping rs2107595 and top LD SNPs (credible set). We have also annotated the rs2107595 SNP as red to make it stand out more clearly for the reader.

**Reviewer #1:** Fig. 6 - when calculating the peak to gene correlation (rs2107595-containing peak vs TWIST1 expression), was the correlation calculation performed within each cell type or across all cell types?

**Response:** Thank you – we agree this is an important point to clarify. The peak to gene correlation method implemented in ArchR corresponds to calculations across all cell types. We have edited the following sentence in the methods section accordingly:

“*After integration, the “getPeak2GeneLinks” function in ArchR was used to correlate chromatin accessibility within peak windows with integrated RNA expression levels across all cell types.*”

**Reviewer #1:** Regarding the association of TWIST1 expression levels with CAD severity and other CAD-related phenotypes - the authors should compare this to a similar analyses with HDAC9 expression.

**Response:** Thank you - we agree this is helpful to add. Associations of HDAC9 expression levels with CAD severity and CAD-related phenotypes were added to Supplementary Table 3 (originally Supplementary Table 5). These data support our argument that HDAC9 has a role in atherosclerosis, because as shown in these new data the levels of HDAC9 are positively associated with the burden of CAD (number of diseased coronary vessels). We modified the text of the manuscript as follows:

Results section: *“Similarly, we also evaluated the phenotypic associations of HDAC9 expression levels in AOR and found that HDAC9 levels were positively associated with the severity of CAD as assessed by number of diseased coronary vessels (P = 0.021), and with a trend also for Duke CAD index (P = 0.06) (Supplementary Table 3).”*

Discussion: *“While our study showed that the HDAC9-associated CAD risk locus does not cause CAD by modulation of HDAC9 levels, structure or function, our study does not suggest that HDAC9 is unimportant in causing atherosclerosis and CAD. On the contrary, this study showed that HDAC9 levels in AOR were positively associated with the burden of CAD (Supplementary Table 3). Consistent with this, our group just completed a separate study showing the importance of Hdac9 in a mouse model of atherosclerosis, whereby knockout of Hdac9 in endothelial cells was associated with reduced burden of atherosclerosis.”*

**Reviewer #1:** Fig. 8 - the authors state that key drivers cannot be determined for GRNs containing >3500 genes, and unfortunately the GRNs derived from the most relevant tissues (AOR and MAM) fit this criterion. Is there any way to modify the GRN parameters so that key drivers can be determined in the relevant tissues?

**Response:** Thank you for this comment. As we have already indicated, the inferring of GRNs and their key drivers from STARNET was a particularly large undertaking that was just published in January 2022 in the inaugural edition of Nature Cardiovascular Research (Koplev et al. A mechanistic framework for cardiometabolic and coronary artery diseases. Nature Cardiovascular Research. 2022;1:85-100). We now cite this manuscript in the current submission. While we therefore believe this comment from Reviewer #1 lies beyond the scope of what is appropriate to address in the context of this manuscript at PLOS Genetics, in brief, the challenge of determining key drivers in large GRNs is essentially an issue of the limitations of the current software (GENIE3) and computational power required for these determinations of key drivers. We specifically mention this issue on page 14 of Koplev et al under the heading “Gene-regulatory coexpression network and key driver inference”, where we state “Due to limitations of GENIE3, only networks from modules with fewer than 3,000 transcripts were inferred.” Accordingly and in response to this comment from Reviewer #1, we have edited this section of our methods section and now state:

*“Key driver analyses were performed using Mergeomics. Note that due to limitations of GENIE3, for large networks (greater than ~3000 genes), it is not possible to infer key drivers.”*

**Reviewer #1:** In the discussion, what is meant by TWIST1 exerting ‘additional’ effects by appearing in a GRN in skeletal muscle? What evidence is there that TWIST1 affects CAD via skeletal muscle, versus simply appearing as a key driver in a skeletal muscle network due to one of the many idiosyncrasies of GRN construction? If the authors wish to assert that TWIST1 affects CAD via skeletal muscle, they should back up this (somewhat unlikely) claim with experimental data.

**Response:** We appreciate this comment. Experimental data to validate these findings would be a very major undertaking. Therefore, while we most respectfully disagree, we acquiesce and have removed this comment from the discussion section. For the sake of clarification only, the impact of metabolic changes on CAD are well known, and our original statement that TWIST1 affects CAD via skeletal muscle was on the basis of the very robust association between this GRN (GRN 116) and the severity of CAD. In particular, GRN 116 is also associated with the severity of CAD as assessed by various parameters including number of coronary lesions, number of diseased vessels and Duke CAD index (Figure 7C). Regardless, as stated, we have removed this comment from the discussion.

**Reviewer #1:** Regarding the consistent directionality of the TWIST1 eQTL and MR results with respect to CAD (points A and B), given that both are derived from the same eQTL resource, these findings are expected to go in the same direction and are not independent validation of one another. Point ‘C’ is nice because it uses a different phenotype (Duke CAD index).

**Response:** Thank you for this comment – we agree. We have edited the text accordingly, which now reads:

“*In the MR analysis TWIST1 was found to be causal for CAD in the arterial wall, again and as expected based on the eQTL results with increased levels of TWIST1 found to promote CAD*.”

**Reviewer #2**

**Reviewer #2:** Ma et al, performed a deep fine mapping of the HDAC9 CAD risk loci and demonstrated, using a succession of bio-informatic tools that the potential causal gene is the region is TWIST1. Furthermore, the authors suggested that the mechanism of action of TWIST1 in CAD might be through the regulation of other genes rather than a direct transcriptional modification of the later. This work is particularly important in highlighting the need of using available data and several bio-informatic tools to explore candidate variants from GWAS before embarking on several experiments that explore the causality and biological implication of GWAS candidates’ loci. Below are my comments.

**Response:** We thank Reviewer #2 for their careful evaluation of our manuscript and positive comments.

**Reviewer #2:** My main concern is about the Mendelian Randomization. Why did the authors choose the sum stat from Nelson et al rather than a much recent and powerfull summary statistic? Also, it is not clear what are the effect estimate used as instruments in the MR. if the lead SNP rs2107595 is significant at the GWAS level and significant for eQTL in AOR and MAM, then using eQTL from STARNET and the GWAS sum statistic violates the principle of MR.

**Response:** Thank you – we appreciate this comment. Nelson (2017) was chosen as it was the most powerful CAD GWAS available at the time of the analysis, with a total sample size of 336,847 individuals available for rs2107595. With respect to effect estimates, we have now provided the following specific new test in the methods section relating to this MR analysis:

“*In terms of effect estimates for this analysis, cis-eQTL data from STARNET was used as per Supplementary Table 1 (effects of rs2107595 on TWIST1 expression in AOR and MAM), while CAD association statistics were obtained from Nelson et al for effects of rs2107595 on CAD risk (variant ID 7:19049388:G:A; effect allele frequency 0.18163; estimate 0.07422; standard error 0.01019; P value 3.41 x 10-13; sample size 336847).”*

To verify MR assumptions, we tested for potential pleiotropy within each tissue using the HEIDI test (implemented in the SMR software). As we state in the results section: “*We also conducted the Heterogeneity In Dependent Instrument (HEIDI) test to further exclude genes with evidence of heterogeneity in their cis-eQTLs. The HEIDI test indicated a significant heterogeneity of effects for MAM (P = 0.001, n=20 SNPs) but not AOR (P = 0.1, n=9 SNPs).”* This finding left only AOR as a suitable candidate for a causal mediation between the rs2107595 variant and the CAD endpoint. This was a very clear and unequivocal result and therefore, we most respectfully disagree that any principles of MR have been violated. Moreover, this finding is entirely logical – in that it makes sense that TWIST1 is causal in the atherosclerosis-prone AOR, but not in the relatively athero-protected MAM. Theoretically, we recognize that it is at least possible that there may still be horizontal pleiotropy at play with rs2107595 simultaneously affecting TWIST1 mRNA levels in MAM and AOR such that this biased the estimated causal effect of TWIST1 in these tissues. However, given the consistency of our findings this appears to be quite unlikely to have had a major impact on our overall data. Nevertheless, we have edited the relevant sentence of the results section to acknowledge this theoretical concern, which now reads:

“*While we cannot fully eliminate the possibility of a minor effect arising from horizontal pleiotropy, we finally estimated a positive causal effect of TWIST1 in AOR (β = 1.87, standard error = 0.26, P = 0.00325), in that increased expression of TWIST1 in AOR is associated with increased risk of CAD*.”

**Reviewer #2:** The authors need to provide the effect estimate of the CAD GWAS and eQTL used for the MR

**Response:** Thank you. We have now provided the following specific new test in the methods section relating to this MR analysis and relevant effect estimates used:

“*In terms of effect estimates for this analysis, cis-eQTL data from STARNET was used as per Supplementary Table 1 (effects of rs2107595 on TWIST1 expression in AOR and MAM), while CAD association statistics were obtained from Nelson et al for effects of rs2107595 on CAD risk (variant ID 7:19049388:G:A; effect allele frequency 0.18163; estimate 0.07422; standard error 0.01019; P value 3.41 x 10-13; sample size 336847).”*

**Reviewer #2:** It will be interesting to also have more insight on the functional prediction of associated SNPs in the locus.

**Response:** Thank you – we gave this comment very careful consideration. We provide a functional assessment of the entire risk locus and all its SNPs in terms of its potential effect on protein structure and function in Figure 3 and the related text in the results section, which showed that none of the SNPs in this locus map to a protein coding region. We also provide eQTL data for all SNPs in this locus, across multiple different tissues (Figure 5, Supplemental Figure 2, and Supplemental Tables 1 and 2). Beyond this, in terms of additional functional prediction of other SNPs and in deciding how best to respond to this comment, we were acutely aware of the fact that one of the main findings of this study is that via several lines of evidence, rs2107595 consistently emerges as the lead SNP and most likely causal variant. We felt that given this finding, it would be of reduced relevance to undertake additional functional assessments of the other SNPs in this locus (beyond the substantial data already provided as above). Moreover, the manuscript is also already quite long (9500 words), with 7 Primary Figures, 4 Supplemental Figures, 1 Primary Table and 3 Supplemental Tables. We felt that adding more data on the other SNPs, especially given that rs2107595 was found to be the causal SNP with a reasonable degree of certainty, would make the manuscript excessively long and might distract readers from the core findings of the study.

**Reviewer #2:** What was the rational in selecting only the lead SNPs for further analysis?

**Response:** Thank you for this comment. As partly discussed above, several factors came into consideration, as follows:

1) We have already evaluated and presented the findings for the effect of all SNPs in this risk locus in terms of protein structure and function (Figure 3).

2) Our eQTL data are for all eQTLs in the risk locus, in all STARNET tissues and also in GTEx (Figure 5, Supplemental Figure 2, and Supplemental Tables 1 and 2). Therefore, based on both #1 and #2, there are already substantive analyses undertaken and presented on all SNPs in this locus.

3) The data supporting the likelihood that rs2107595 is the lead/causal SNP are consistent and appear to be robust. While not entirely discounting the possibility that another SNP might be causal, this nevertheless diminishes the relevance of the further analyses of other SNPs.

4) The overarching objective of the manuscript was to understand at a wholistic level how this risk locus causes CAD, and to resolve prior controversies about the importance of HDAC9 versus TWIST1 in mediating the effects of this locus. We believe we have very successfully accomplished these objectives. We did not aim to perform exhaustive studies to map all the different possibilities for how each SNP in the locus might theoretically cause CAD. Such extended functional studies are beyond the scope of this manuscript.

5) The manuscript is also already quite long (9500 words), with 7 Primary Figures, 4 Supplemental Figures, 1 Primary Table and 3 Supplemental Tables.

6) We are concerned that adding more analyses of non-lead SNPs (when there is a clear lead SNP), for a manuscript that has very successfully achieved its primary objectives but which is already quite long, runs the risk of distracting its readers or making the manuscript too unwieldy/tangential to readily understand.

**Reviewer #2:** Titles “ An Efficient Informatics Pipeline to Understand Complex Gene Loci: The HDAC9- associated risk locus promotes coronary artery disease by governing TWIST1” read as the authors are presenting a pipeline that they develop in the current paper however, it seem like the pipeline have already been published and this work is just a continuation. The title should be modified accordingly.

**Response:** We appreciate this comment from Reviewer #2. We have amended the title, which now is simply “The HDAC9-associated risk locus promotes coronary artery disease by governing TWIST1”.

**Reviewer #2:** Table 1: the authors need to clarify the source of the effect size in the table 1 and to which diseases (I suppose CAD) the effect size corresponds given that some SNPs have been reported in multiple phenotypes.

**Response:** Thank you - this is important to clarify. In table 1, none of the SNPs had a stronger beta and/or smaller P value for atherosclerotic aortic calcification (AAC) or stroke than for CAD, and therefore the beta, standard error and P value listed in Table 1 are for CAD. We have added this exact text to the legend for Table 1:

*“None of the SNPs had a stronger beta and/or smaller P value for AAC or stroke than for CAD, and therefore the beta, standard error and P value listed in Table 1 are for CAD.”*

**Reviewer #2:** Figure 2: The authors should create a full LDheatmap (R2 between 0 and 1 for all SNPS include in the plot) that display the correlation for all SNPs; the current map display only R2>0.4 or R2>0.8 this is misleading as it doesn’t clearly show the full picture of LD between SNPs. WE expect to see a single block as the authors suggested.

**Response:** Thank you for this suggestion. We agree this is a better way to represent these LD relationships. Rather than the original 2 separate LD heatmaps for R2 > 0.8 and R2 > 0.4, we now present only the single full LD heatmap displaying the correlations among all SNPs in Figure 2. As expected, this shows only a single block, consistent with there being only a single causative SNP.

**Reviewer #2:** ST1and ST2 should also have the results for HER1 and HDAC9 for comparison

**Response:** We appreciate this comment, which indicates to us that we have inadequately explained these tables. For clarity, we assume the Reviewer #2 has made a typographical error and rather than HER1, meant *FERD3L*. Supplementary Table 1 using STARNET, and Supplementary Table 2 which is the identical analysis in GTEx, are showing all cis eQTLs related to the *HDAC9*-associated risk locus (within 1 MB upstream or downstream of the *HDAC9*-associated locus). The reason ST1 and ST2 do not include *HDAC9* or *FERD3L*, is because there were no eQTLs related to the expression levels of either of these genes. So in fact, the results or *FERD3L* and *HDAC9* were included in these tables already – the result being there were no eQTLs for these genes.

We have clarified this in the revised manuscript now explicitly state in the legends to Supplementary Tables 1 and 2 that: “*There were no cis eQTLs identified for HDAC9 or FERD3L.*”

**Reviewer #2:** ST5 has a typos in the title “Supplementary Table5. Phenotypic associatinos of TWIST1 expression levels in AOR in STARNET subjects”

**Response:** Thank you – this has been corrected (original Suppl Table 5 is now Suppl Table 3).
